# Development of a checklist to validate the framework of a narrative medicine program based on Gagne’s instructional design model in Iran through consensus of a multidisciplinary expert panel

**DOI:** 10.3352/jeehp.2019.16.34

**Published:** 2019-10-31

**Authors:** Saeideh Daryazadeh, Nikoo Yamani, Payman Adibi

**Affiliations:** 1 Department of Medical Education and Medical Education Research Center, Medical Education Development Research Center, Isfahan University of Medical Sciences, Isfahan, Iran; 2 Department of Internal Medicine, Research Center of Gastroenterology, Isfahan University of Medical Sciences, Isfahan, Iran; Hallym University, Korea

**Keywords:** Narrative medicine, Teaching, Educational models, Assessment, Program development, Iran

## Abstract

**Purpose:**

Narrative medicine is a patient-centered approach focusing on the development of narrative skills and self-awareness that incorporates “attending, representing, and affiliating” in clinical encounters. Acquiring narrative competency promotes clinical performance, and narratives can be used for teaching professionalism, empathy, multicultural education, and professional development. This study was conducted to develop a checklist to validate the framework of a narrative medicine program through consensus of a panel.

**Methods:**

This expert panel study was conducted from 2018 to 2019 at Isfahan University of Medical Sciences, Iran. It included 2 phases: developing a framework in 2 steps and forming an expert panel to validate the framework in 3 rounds. We adapted a 3-stage narrative medicine model with 9 training activities from Gagne’s theory, developed a framework, and then produced a checklist to validate the framework in a multidisciplinary expert panel that consisted of 7 experts. The RAND/UCLA appropriateness method was used to assess the experts’ agreement. The first-round opinions were received by email. Consensus was achieved in the second and third rounds through face-to-face meetings to facilitate interactions and discussion among the experts.

**Results:**

Sixteen valid indicators were approved and 100% agreement was obtained among experts (with median values in the range of 7–9 out of a maximum of 9, with no disagreement), and the framework was validated by the expert panel.

**Conclusion:**

The 16 checklist indicators can be used to evaluate narrative medicine programs as a simple and practical guide to improve teaching effectiveness and promote life-long learning.

## Introduction

### Background/rationale

The narrative approach is a learning method used to enhance the meaning and experiential aspect of medical education, and is widely used when recalling, imagining and retelling past experiences [1,2]. Learning through narratives includes changes in knowledge and attitudes. Rita Charon at Colombia University introduced “narrative medicine” (NM) in 2000 as a clinical framework that improves “scholarship, education, and clinical practice,” as well as fostering “empathy, reflection, professionalism, and trustworthiness” [3]. NM is a patient-centered approach focusing on the development of narrative skills and self-awareness that incorporates “attending, representing, and affiliating” in clinical encounters. “Attention” revolves around a patient’s visit and the interaction of the clinician with the patient, “representation” refers to a physician’s understanding of a patient’s illness as articulated in written form, and “affiliation” refers to deep reflections that are analyzed and shared with colleagues in the form of narratives. The use of narrative skills in clinical practice is essential in order to diagnose and treat diseases. Narrative skills include “reading, writing, and attending” in illness stories that are components of reflective training. Reflective training improves trainees’ understanding of patients’ culture, increases their commitment to colleagues, and enhances the effectiveness of the healthcare team [4]. In addition, narratives can be used for teaching professionalism, empathy, multicultural education, and other aspects of medical education [5]. Therefore, NM has been introduced as a valuable tool in medical education and constitutes an approach to “professional development” in medicine [6].

NM is being taught more frequently at many universities around the world, with different educational aims and within the framework of various programs. By the same token, its educational effectiveness has been examined in previous studies [6]. Although some educational guides have been provided for reflective training, providing an educational framework with guidelines for how to implement an NM course would be useful, especially for universities with no experience with NM that intend to implement NM as an innovative teaching method.

### Purpose

This study was conducted to develop a checklist to validate the framework of an NM program based on Gagne’s instructional design model in Iran through consensus of a multidisciplinary expert panel. The specific goals were as follows: first, the framework for an NM program was developed; second, a checklist to validate the framework of NM was suggested; and third, the checklist was tested by an expert panel for consensus.

## Methods

### Ethics statement

This research was part of a project with the ethics code IR.MUI.REC.1396.3.472 at Isfahan University of Medical Sciences in Iran. Informed consent was obtained from the panel members.

### Study design

This was a modified Delphi expert panel study that included 2 phases: (1) developing the framework, and (2) forming an expert panel to achieve consensus regarding the framework. This study took a year (September 2018 to September 2019) to review the literature and to design, implement, and finalize the framework.

### Expert panel participants

Seven experts were recruited by purposive sampling to participate in the expert panel. Their specialties were medical education, curriculum development, instructional design, internal medicine, psychiatry, and narrative analysis. All of the participating specialists were from Iran, and all participants were familiar with NM. Due to the interdisciplinary nature of NM, experts were selected who had multidisciplinary and interdisciplinary specialties (Supplement 1).

### Setting

#### Phase I: development of the framework of an NM program

First step: We developed an initial draft of the NM framework based on a literature review of the 3-stage NM model, as well as our experiences of conducting this course, and we allocated these 3 stages to 2 theoretical and practical training sections.

NM model: The NM model included reading a narrative (“attending”), reflective writing (“representing”), and small-group discussions and sharing experiences with others (“affiliating”) [7]. We adapted the NM model grounded in Gagne’s theory and developed a draft of the framework.

Gagne’s instructional design model: Gagne explained that learning levels include verbal knowledge and memory, mental ability, cognitive approaches, performance ability, and emotional beliefs, and that learning objectives should be presented at these levels. Gagne’s theory has 3 components, including learning results, the circumstances of learning, and a set of 9 training activities. Interior and exterior learning circumstances through specific learning results are realized in training activities. In each training activity, particular actions are taken to achieve the training results [8]. Gagne’s theory derives from the theory of behavioral learning, and is one of the most popular models of instructional design.

We chose Gagne’s theory in order to match the 9 training activities with the steps of the NM model. In this model, the details of training activities tailored to the NM sessions were well addressed. Furthermore, this approach emphasizes educational effectiveness through participatory learning in small groups and life-long learning, a theme that is also highlighted in NM.

Therefore, we integrated the 3-stage NM model used for reflective training with Gagne’s theory that included 9 steps of training activities (Supplement 2).

Second step: We developed a checklist containing indicators of instructional design components to validate the framework (Table 1). Next, we utilized a scale with scores ranging from ‘completely disagree’=1 to ‘completely agree’=9 (range, 1–9) to measure responses, following the RAND/UCLA appropriateness method (RAM) developed by the RAND Corporation and the University of California Los Angeles for ranking indicators. The criteria for reaching agreement (consensus) on each indicator were set as follows: 1–3 as “inappropriate,” 4–6 as “uncertain,” and 7–9 as “appropriate” [9,10].

(1) Agreement: If the scoring range of all indicators was in a single one of the intervals (1–3, 4–6 and 7–9), agreement was attained. (2) Disagreement: If the scoring range was distributed across all 3 of the intervals (1–3, 4–6, and 7–9), then there was disagreement. (3) Acceptability: Acceptability was defined as a median score in the range of 7–9 with no disagreement. Otherwise, an indicator was considered to be inappropriate.

#### Phase II: expert panel

To validate the framework of NM, we formed an expert panel. The panel consisted of multidisciplinary experts who were qualified to comment on NM training. Furthermore, we incorporated instructional design into this process. The framework approval process was conducted in 3 rounds. We applied RAM, which is a modified Delphi method, to determine the agreement of experts [10]. This method uses a combination of Delphi techniques (mailed questionnaires), and nominal group techniques (face-to-face sessions); furthermore, it is a dynamic process, as in addition to presenting a clear criterion for ranking the indicators, it takes advantage of group interactions and discussions accordingly [9]. Therefore, we used this method to avoid any ambiguity and the possibility of ignoring important indicators or details.

We performed the first round through email. The second and third rounds were conducted through face-to-face meetings to promote interactions and discussion among the experts, and the interval between each round was 2 weeks.

First round (email): We explained the purpose of the NM educational framework to each of the 7 experts separately and asked them to participate in the expert panel for validating the framework. After the experts agreed to collaborate, we sent them the primary draft along with the checklist via email detailing the ranking criteria. The framework and the checklist were given to the experts with explanations of how to rank the indicators. We asked the experts to rank the criteria in the draft version based on the indicators listed in the checklist and to present their comments on ways to supplement and improve the framework at the next meeting.

Second round (face to face): We held a face-to-face meeting with 7 experts, and a moderator guided the panel and facilitated group discussions in order to gather the experts’ comments. At the beginning of the session, we provided an overview of the goal of formulating the framework and ranking the criteria in the checklist. In this session, we compared the ratings for each indicator in the first round and addressed them in a group discussion and recorded the experts’ suggestions on each indicator. Then, we revised the indicators according to the experts’ comments (Tables 2, 3). After revising the draft, we sent the edited version to the experts and asked them to present their ratings and recommendations again. In addition, we invited them to attend a face-to-face meeting in the third round.

Third round (face to face): We held the second face-to-face meeting to finalize the draft. The 7 experts suggested minor changes and finally consensus was achieved among the experts. We extracted the median, minimum, and maximum ratings for each indicator. In the second round, we corrected the ambiguities pointed out in the first round, and in the third round the draft was validated with minor edits to the wording.

**Fig. 1.**
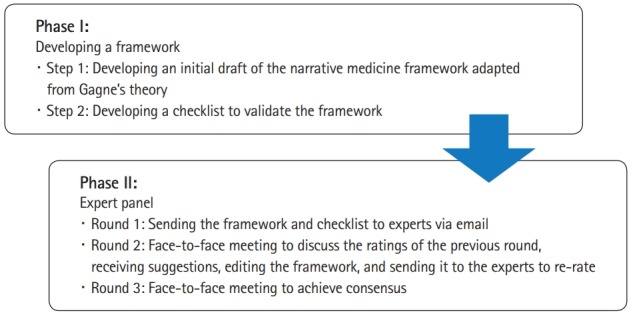
Flow diagram of this panel study.

The median of the ratings was calculated to determine the level of agreement among the experts for each indicator in the checklist. The flow diagram of this panel study is presented in Fig. 1.

### Statistics

The median of the ratings was calculated using IBM SPSS ver. 23.0 (IBM Corp., Armonk, NY, USA).

## Results

### The educational framework

The educational framework incorporating NM was designed based on the 3 components of Gagne’s theory. This framework includes learning outcomes, learning circumstances, and training activities. A summary of this program concerning teaching professionalism is provided in Supplements 3 and 4. The recommended program based on the 9 training activities in Gagne’s theory encompassed learning stages, training activities, instructions, media, and teaching methods (Supplement 4).

### Consensus among the experts

The experts agreed on 16 indicators after the third round. In the first round, 14 indicators were sent to experts via email. In the second round, there were differences in the ratings of the indicators in the first round. After the group discussion, the experts suggested changing 2 indicators, making minor modifications, and adding 2 new indicators. In the third round, 16 indicators were rated by experts and the framework was confirmed with minor changes in writing (Table 4). Of note, indicators 1 and 3 did not exist in the first round; they were recommended in the second round by the experts and were added to the checklist for the third round (final checklist in Table 2). In the second round, the experts only gave comments to improve the framework, and after revision, they confirmed it in the third round. Finally, 16 valid indicators for the framework were approved and 100% agreement was obtained among the experts (median values in the range of 7–9, with no disagreement).

## Discussion

### Key results

We conducted this study to develop a framework to teach and evaluate an NM program based on Gagne’s theory and to develop a validation tool by obtaining consensus in the expert panel. We adapted the 9 training activities in Gagne’s theory for the 3-stage NM model. The experts reached full agreement after 3 rounds and approved the framework. This framework encompasses learning results based on interior and exterior learning circumstances and 9-step learning activities in NM. This framework can provide a scaffold for initial learning at universities in Iran that intend to present an NM program, and can serve as a practical guide for faculty members who are planning to implement an NM course for the first time. Since NM is an innovative teaching method in medical education, and subject matter specialists are also limited, this structured program will be helpful for medical instructors. The 16 checklist indicators to validate the framework of an NM program constitute a meaningful innovation in this field. This tool was also agreed upon by a panel, so that it can be used to evaluate an NM program.

### Comparison with other research

Various universities have provided some rubrics or reflective training guides to improve reflection [4,11,12]. Charon and colleagues provided a reflective writing guide to teach reflection through creative writing and close reading that included “reading, writing, and attending to the stories of illness.” Creative writing emanates from theories of aesthetics and emphasizes understanding, description, and acceptance [4]. Accordingly, some studies used 3 stages (reading, reflection, and group discussion), while others applied 2 stages (reading or reflection and group discussion) for teaching NM [6]. We also developed and applied a 3-stage model for the NM framework.

Applying reflection in medicine is effective for improving communication skills, professionalism, critical thinking, clinical knowledge, practical understanding, clinical reasoning, and the reduction of medical and diagnostic errors [11]. Therefore, reflective training is an important part of medical education. Accordingly, students’ reflective capacity should be evaluated, given the value of reflection for life-long and clinical decision-making [13]. The REFLECT rubric is a practice guide developed by Wald et al. [11] for reflective writing training. Due to the clear explanation of reflection levels and writing components in the REFLECT rubric, we recommend using this tool in reflective training and applying it both for teaching and evaluating reflective capacity.

Furthermore, it is essential for educators to provide effective feedback in order for students to develop their level of reflection. For this purpose, a comprehensive and practical tool called BEGAN was designed by Reis and colleagues at Brown University as a practical guide for teachers to foster reflective capacity in reflective writing [14]. We recommend using a feedback guide such as the Pendleton Rules to provide feedback [15]. It is worth noting that NM has 2 important components: teaching guided reflection and providing constructive feedback to learners. These components are essential to improve reflective capacity.

### Limitations

Because NM was first implemented in Iran at Isfahan University of Medical Sciences, we were limited in the selection of experts in the field in terms of their institutional background. However, the expert panel also included 3 specialists (medical education, internal medicine, and psychiatry) who had spent a part of their professional career or academic education at universities outside of Iran (including the United States, Sweden, and Germany) and were familiar with the subject of NM. To minimize this limitation, we included multidisciplinary experts who examined the different dimensions of the framework.

### Conclusion

In clinical education, NM can be used as a teaching method and as a basis for teaching reflection and improving core competencies in medicine. This study provided an explicit and clear framework for describing the process of teaching NM, adapted with an instructional design model as a structured program. Additionally, reflective training in the NM model is highly compatible with the training activities presented in Gagne’s theory. The importance of this framework lies in its applicability and ease of use by medical teachers for educational goals. Furthermore, adapting Gagne’s theory to apply to the NM model is a positive aspect of this study because of its impact on enhancing the quality and effectiveness of teaching and promoting life-long learning. This framework can serve as a guide for educational departments at medical universities to implement NM programs. The checklist presented in this study can be used widely to evaluate the framework of NM courses.

## Figures and Tables

**Table 1.** Initial checklist

No. of indicator	Gagne’s instructional design components	Appropriateness criteria								
		Inappropriate			Uncertain			Appropriate		
		1	2	3	4	5	6	7	8	9
1	Cognitive approaches									
2	Performance abilities									
3	Emotional beliefs									
4	Exterior circumstances									
5	Interior circumstances									
6	Step 1. Attracting and attending									
7	Step 2. Raising learners’ awareness of objectives									
8	Step 3. Evoking memory of past knowledge									
9	Step 4. Presenting training materials									
10	Step 5. Providing a guide for learning									
11	Step 6. Examining performance									
12	Step 7. Giving feedback									
13	Step 8. Evaluating performance									
14	Step 9. Improving and transferring learning									

**Table 2.** Final checklist

No. of indicator	Gagne’s instructional design components			Appropriateness criteria								
				Inappropriate			Uncertain			Appropriate		
				1	2	3	4	5	6	7	8	9
1	Before training	Learning results	Mental abilities									
2			Cognitive approaches									
3			Verbal knowledge and memory									
4			Performance abilities									
5			Emotional beliefs									
6		Learning circumstances	Exterior									
7			Interior									
8	Training	9 Training activities	Step 1. Attracting and attending									
9			Step 2. Raising learners’ awareness of objectives									
10			Step 3. Evoking memory of past knowledge									
11			Step 4. Presenting training materials									
12			Step 5. Providing a guide for learning									
13			Step 6. Examining performance									
14			Step 7. Giving feedback									
15			Step 8. Evaluating performance									
16	After training		Step 9. Improving and transferring learning									

**Table 3.** Recommendations of the experts in round 2

No.	Indicator in round 1	Recommendations of the experts in round 2
1	Cognitive approaches	Separate the learning results related to the cognitive domain based on Gagne’s instructional design model.
		Add 2 indicators (mental abilities, and verbal knowledge and memory).
		Place learning circumstances based on learning results in a separate table.
2	Performance ability	Place learning circumstances based on learning results in a separate table.
3	Emotional beliefs	Previous recommendation
4	Exterior circumstances	Previous recommendation
5	Interior circumstances	Previous recommendation
6	Step 1. Attracting and attending	In writing the instructions for steps 1 to 9, make minor edits to make the wording easy to understand for clinical teachers.
7	Step 2. Raising learners’ awareness of objectives	Previous recommendation
8	Step 3. Evoking memory of past knowledge	Previous recommendation
9	Step 4. Presenting training materials	Previous recommendation
10	Step 5. Providing a guide for learning	Previous recommendation
11	Step 6. Examining performance	Previous recommendation
12	Step 7. Giving feedback	Previous recommendation
13	Step 8. Evaluating performance	Previous recommendation
14	Step 9. Improving and transferring learning	Previous recommendation

**Table 4.** The median scores for each indicator

No.	Indicator	Median of round 1 (min–max)	Median of round 3 (min–max)
1	Mental abilities	-^a)^	9 (7–9)
2	Cognitive approaches	6 (3–8)	9 (7–9)
3	Verbal knowledge and memory	-^a)^	9 (7–9)
4	Performance ability	7 (3–8)	9 (7–9)
5	Emotional beliefs	8 (3–8)	9 (7–9)
6	Exterior circumstances	8 (3–8)	9 (8–9)
7	Interior circumstances	6 (3–8)	9 (8–9)
8	Step 1. Attracting and attending	8 (5–9)	9 (7–9)
9	Step 2. Raising learners’ awareness of objectives	8 (7–9)	9 (7–9)
10	Step 3. Evoking memory of past knowledge	8 (7–9)	9 (7–9)
11	Step 4. Presenting training materials	8 (5–9)	9 (8–9)
12	Step 5. Providing a guide for learning	8 (6–9)	9 (8–9)
13	Step 6. Examining performance	8 (6–9)	9 (7–9)
14	Step 7. Giving feedback	8 (7–9)	9 (7–9)
15	Step 8. Evaluating performance	8 (7–9)	9 (8–9)
16	Step 9. Improving and transferring learning	8 (6–9)	9 (8–9)

a) This indicator did not exist in the first round.

## Supplementary materials

Data files are available from https://doi.org/10.7910/DVN/KWN3TU

Supplement 1. Gender and expertise of panelists

Supplement 2. Adaptation of Gagne’s instruction theory with the narrative medicine model

Supplement 3. Learning circumstances based on learning results

Supplement 4. Teaching and assessing in reflective training based on the 9 training activities in Gagne’s theory

Supplement 5. Audio recording of the abstract.
