# Supplementary material for: Development of a checklist to validate the framework of a narrative medicine program based on Gagne’s instructional design model in Iran through consensus of a multidisciplinary expert panel
Source: J Educ Eval Health Prof. 2019 Oct 31;16:34. doi: 10.3352/jeehp.2019.16.34 (PMC6895376; doi:10.3352/jeehp.2019.16.34)
Supplement: Supplementary file 2 — Supplement 1. Gender and expertise of panelists [file jeehp-16-34-suppl1.pdf]

Jeehp

Supplement 1. Gender and expertise of panelists

No. of experts	Gender (male/female)	Profession	Area of expertise
2	Female, female	Faculty member, associate professor	Curriculum development, instructional design, medical education
1	Male	Faculty member, professor	Gastroenterology, internal medicine, medical education
1	Male	Faculty member, professor	Psychiatry
2	Male, female	Faculty member, assistant professor	Medical education
1	Female	MSc, MD, narrative analyst	Medical education, general physician
