# Supplementary material for: Development of a checklist to validate the framework of a narrative medicine program based on Gagne’s instructional design model in Iran through consensus of a multidisciplinary expert panel
Source: J Educ Eval Health Prof. 2019 Oct 31;16:34. doi: 10.3352/jeehp.2019.16.34 (PMC6895376; doi:10.3352/jeehp.2019.16.34)
Supplement: Supplementary file 3 — Supplement 2. Adaptation of Gagne’s instruction theory with the narrative medicine model [file jeehp-16-34-suppl2.pdf]

Supplement 2. Adaptation of Gagne's instruction theory with the narrative medicine model

Stages of learning	Training activities	Reflective training
Readiness for learning (realization of interior circumstances)	Step 1. Attracting and attending	Attending: reading a narrative or artwork such as a poem
	Step 2. Raising learners' awareness of objectives	
	Step 3. Evoking memory of past knowledge	
Performance (realization of exterior circumstances)	Step 4. Presenting training materials	Representing: reflective writing
	Step 5. Providing a guide for learning	
	Step 6. Examining performance	Affiliating: responding (small group discussion and sharing experiences with others)
	Step 7. Giving feedback	
Transmission of learning (realization of exterior circumstances)	Step 8. Evaluating performance	
	Step 9. Improving and transferring learning	
