# Supplementary material for: Development of a checklist to validate the framework of a narrative medicine program based on Gagne’s instructional design model in Iran through consensus of a multidisciplinary expert panel
Source: J Educ Eval Health Prof. 2019 Oct 31;16:34. doi: 10.3352/jeehp.2019.16.34 (PMC6895376; doi:10.3352/jeehp.2019.16.34)
Supplement: Supplementary file 4 — Supplement 3. Learning circumstances based on learning results [file jeehp-16-34-suppl3.pdf]

Supplement 3. Learning circumstances based on learning results

Learning results	Learning circumstances	
	Interior	Exterior
Mental abilities	Identifying reflection levels in a reflective narrative	Explaining reflection levels and parts of a reflective narrative
	Learning the concepts and principles of reflective narrative	Providing the concepts and principles of reflection and narrative writing through the educational tool of reflective practice
	Ability to apply concepts and rules to reflective writing	
Cognitive approaches	Recalling clinical experiences of encountering with patients	Providing a reflective narrative without analyzing it
	Considering reflection levels and writing components	Expressing clinical experience as a reflective narrative
Verbal knowledge and memory	Recalling meaningful and relevant information from previous learning	Providing information on new topics such as definitions of NM, narration, reflection, empathy, and expressing the relation of NM to professionalism
	Thinking about the importance of professionalism and its impact on clinical practice	
Performance ability	Learning to write a reflective narrative by separating the components of a narrative and the reflection levels	Training in narrative writing and explaining the components of a narrative according to reflection levels

NM, narrative medicine.
