# Supplementary material for: Development of a checklist to validate the framework of a narrative medicine program based on Gagne’s instructional design model in Iran through consensus of a multidisciplinary expert panel
Source: J Educ Eval Health Prof. 2019 Oct 31;16:34. doi: 10.3352/jeehp.2019.16.34 (PMC6895376; doi:10.3352/jeehp.2019.16.34)
Supplement: Supplementary file 5 — Supplement 4. Teaching and assessing in reflective training based on the 9 training activities in Gagne’s theory [file jeehp-16-34-suppl4.pdf]

Supplement 4. Teaching and assessing in reflective training based on the 9 training activities in Gagne's theory

Session	Stages of learning	Training activities	Instructions	Media and teaching methods
Theoretical	Readiness for learning (realization of interior circumstances)	Step 1. Attracting and attending	Performing an initial evaluation by asking to write a reflective narrative from learners	Educational clips, PowerPoint slides
			Questioning learners about their interest in reading books or poems, watching movies, and writing diaries	Lecture, question and answer, advance-organizers' presentation
			Screening a short clip about the related topic (like empathy with a patient or professionalism) to engage and motivate learners	
		Step 2 Raising learners' awareness of objectives	Presenting learning objectives and expressing educational expectations at the end of the course	
		Step 3. Evoking memory of past knowledge	Asking learners about the need for professionalism and expressing experiences of empathy with patients	
	Performance (realization of exterior circumstances)	Step 4. Presenting training materials	Organizing the sequence of presenting educational content in a rational and comprehensible manner to learners	PowerPoint slides, reflective training tools, training package including articles and educational pamphlets
			Reading examples of written narrative and providing theory training	Lecture, question and answer, group discussion, role-playing, participatory learning, flipped classroom
			Introducing a reflective educational tool (such as the REFLECT tool [10])	
Practical		Step 5. Providing a guide for learning	Teaching narrative writing and reflective narrative analysis based on reflection tool	
			Forming small groups of 6-8 people	
			Practicing reflective narrative writing with the considered theme	
		Step 6. Examining performance	Analyzing learners' narratives based on reflection levels, formative assessment	
		Step 7. Giving feedback	Discussing in small groups and providing feedback from peers, clinical facilitator, and a narrative analyst on reflective narratives	
	Transmission of learning (realization of exterior circumstances)	Step 8. Evaluating performance	Writing reflective narratives by learners	Helping to transfer and apply the learning in the real world by suggesting that learners write reflective narratives in a logbook or portfolio
			Assessing narratives based on a reflection tool to determine the "reflective capacity" of learners, summative assessment	
			Assessing learners using related professionalism tools	
		Step 9. Improving and transferring learning	Applying narrative medicine in clinical settings	
